# Treatment of Drug-Resistant Epilepsy With Right-Sided Vagus Nerve Stimulation

**DOI:** 10.7759/cureus.65061

**Published:** 2024-07-21

**Authors:** Yitao Ma, William Young

**Affiliations:** 1 Neurology, Walter Reed National Military Medical Center, Bethesda, USA

**Keywords:** vagus nerve stimulation, cardiac complication, long-term outcome, off-label use, drug-resistant epilepsy

## Abstract

Vagus nerve stimulation (VNS) has been used as an adjunctive therapeutic option for drug-resistant epilepsy for decades. Traditionally, the left vagus nerve is used for stimulation, while the right vagus nerve is rarely used. The long-term efficacy and safety of the right VNS (R-VNS) in humans are unknown. We presented three patients who were treated with R-VNS over a follow-up period of up to eight years. All three patients tolerated R-VNS well with minimal complications. R-VNS displayed reasonable effectiveness in all three patients. One patient had an excellent response and became seizure-free. The other two patients demonstrated a less favorable response to R-VNS compared to their previous left VNS therapy.

## Introduction

Epilepsy is a common and debilitating neurological disorder that affects 1-2% of people around the world [1]. About one-third of these patients develop drug-resistant epilepsy (DRE) after failing to achieve seizure freedom with adequate trials of two anti-seizure medications (ASMs) [2]. These patients normally require an epilepsy surgery evaluation to decrease their seizure burden. However, many patients are also not good candidates for surgical resection due to seizure onset zones being multifocal or affecting the eloquent cortex. For this group of patients, neuromodulation such as vagus nerve stimulation (VNS), responsive neurostimulation, and deep brain stimulation can be considered to improve seizure control. Left VNS (L-VNS) was approved by the US Food and Drug Administration (FDA) as an adjunct treatment for DRE in 1997 [3]. Multiple studies have proven L-VNS to be a safe and effective treatment with few complications [4]. Compared to other medical devices, VNS is the most cost-effective therapy [5]. Over the years, VNS has emerged as a valuable treatment option for patients with DRE.

The VNS system includes a programmable, lithium battery-powered pulse generator implanted in the left chest. It is connected to a bipolar lead wrapping around the left vagus nerve to deliver electric impulses to the brain [6]. Due to the presumed risk of stimulation-induced cardiac arrhythmia, the left side is the preferred side for stimulation [7]. When L-VNS is not technically feasible, the right VNS (R-VNS) has rarely been used, which has shown favorable short-term outcomes [8-10]. R-VNS is an off-label use. The long-term safety and efficacy of R-VNS in humans are still unknown. We presented three patients with R-VNS in our center over a follow-up period of eight years.

## Case presentation

This study was approved by the Institutional Review Board of Walter Reed National Military Medical Center. Three consecutive patients who had R-VNS to treat DRE between 2000 and 2023 were identified. The clinical information of the patients was extracted from the electronic medical record system. We aim to study the long-term outcome and safety profile of R-VNS. VNS outcomes were measured by the McHugh classification system [11].

The first patient is a 52-year-old woman with static encephalopathy who started to have frequent seizures with cognition decline at age five. Multiple seizure types, including absence seizures, atonic seizures, focal motor seizures, and generalized tonic-clonic seizures (GTCs), were reported by family members. She became seizure-free at age eight and went many years with good seizure control, except for occasional focal seizures. Her seizures then returned at age 38, mainly as focal motor seizures with impaired awareness and focal to bilateral tonic-clonic seizures (FBTCs). She had about five to six seizures per month, with an increasing frequency triggered by various life stressors. Long-term EEG monitoring captured nine left frontotemporal-onset clinical and electrographic seizures. After failing multiple ASMs, she had L-VNS implanted in 2012. She responded well to VNS, with seizure reductions of approximately 50-60%. VNS eliminated all her atonic seizures. However, the entire VNS system had to be removed in 2015 due to lead breakage. In August 2016, a new generator was implanted in her left chest with leads applied to the right vagus nerve for stimulation due to extensive scarring of the left one. No perioperative or postoperative cardiac complications were noted. She still had four to five seizures per year initially but has been seizure-free in the past three years after her VNS was programmed to the optimum settings.

**Figure 1:**
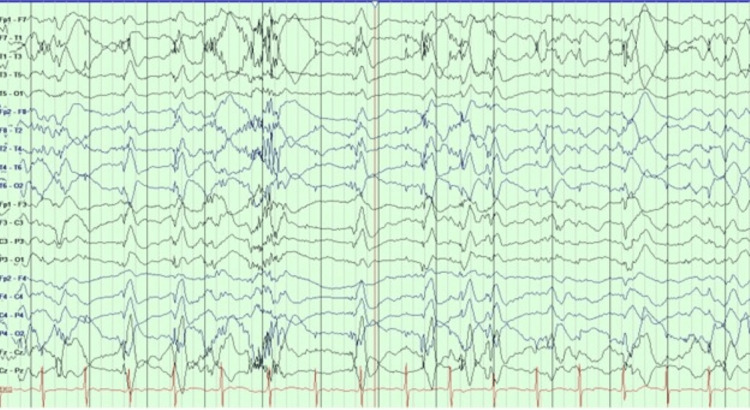
EEG showed generalized 1.5-2 Hz spike and wave discharges EEG: electroencephalogram

**Figure 2:**
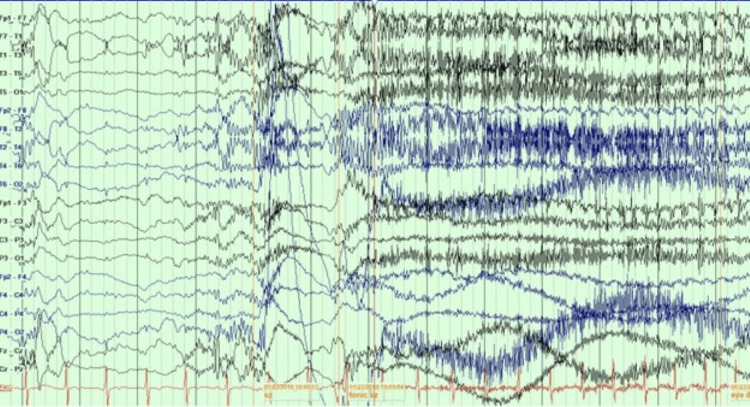
EEG showed a tonic seizure associated with generalized PFA EEG: electroencephalogram, PFA: paroxysmal fast activity

The second patient is a 42-year-old man with Lennox-Gastaut syndrome (LGS). He started to have seizures at nine months old after viral meningoencephalitis. He suffered multiple seizure types, with the most debilitating one being FBTC with ictal cyanosis. He had about 12-23 seizures per month. EEG showed generalized 1.5-2 Hz spike and wave discharges with moderate generalized slowing (Figure 1) and tonic seizures associated with generalized paroxysmal fast activity (PFA) (Figure 2).

Primary L-VNS was implanted in 2002, with a significant reduction in seizures. However, wire breakage and device failure occurred in 2005 due to his striking behavior. Replacement of the device was complicated by a staphylococcus infection requiring intravenous antibiotic treatment. The entire generator was then removed, leaving electrodes attached to the left vagus nerve. His seizure frequency increased significantly with the device failure. Initially, his parents hesitated to have the VNS system reimplanted because of possible recurrent infections. They eventually decided to pursue it in 2018 due to his high seizure burden. The neurosurgery team determined it was not possible to place electrodes distal to existing electrodes and implanted a right-sided generator with electrodes attached to the right vagus nerve. Test stimulation was performed intraoperatively without reflex bradycardia. He tolerated R-VNS well without any signs of infection or discomfort after surgery but developed prolonged cyanosis with FBTCs when the output current was programmed to 1.5 mA. His setting was then decreased to 1.375 mA without further similar events. He still had 8-16 seizures per month with R-VNS. His FBTCs are less frequent and intense, with a shorter postictal state. He went on to have a complete corpus callosotomy two years later, and his seizures have been under good control. He currently has three to four seizures per month. Multiple rounds of continuous EKG monitoring during subsequent inpatient and outpatient surgical procedures demonstrated no clinically significant cardiac arrhythmia.

**Figure 3:**
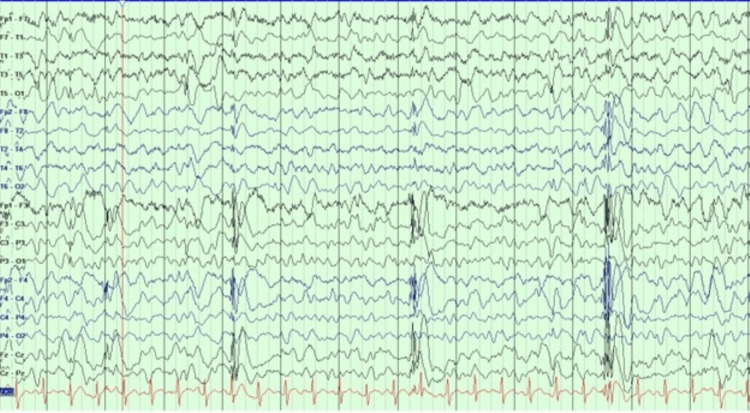
EEG showed generalized 1.5-2 Hz spike and wave discharges and multifocal spikes EEG: electroencephalogram

The third patient is a 20-year-old man who developed epileptic spasms at age three. He was treated with corticosteroids, but later his ES evolved into LGS. He was having multiple seizures daily. His FBTCs often last over 10 minutes. His EEG showed a generalized 1.5-2 Hz spike, wave discharges, and multifocal sharps (Figure 3).

L-VNS was placed at age four. His parents reported a substantial seizure reduction of more than 50%, and he stabilized at two to four FBTCs per month. His VNS was complicated with recurrent infections. The generator was removed in July 2020, which led to increased seizure frequency. He had R-VNS implanted four months later, after his infection cleared up. No arrhythmias were observed intraoperatively during the standard lead test. With his R-VNS, he has been having more frequent FBTCs, up to seven per month, even though his output current was titrated at 2.25 mA and duty cycle was up to 41%. He tolerated R-VNS well with no complications. The clinical information of all the patients is summarized in Table 1.

**Table 1:** Summary of clinical information of all patients ID: intellectual disability, GDD: global developmental delay, GSSW: generalized slow spike and wave, ASMs: anti-seizure medications, VPA: valproic acid, LEV: levetiracetam, CBZ: carbamazepine, CBD: cannabidiol, CLB: clobazam, RUF: rufinamide, LTG: lamotrigine, ZNS: zonisamide, L-VNS: left vagus nerve stimulation, R-VNS: right vagus nerve stimulation, LGS: Lennox-Gastaut syndrome

Variables	Patient 1	Patient 2	Patient 3
Age (yrs), gender	53, F	42, M	20, M
Age at epilepsy onset (yrs)	5	0.75	3
Comorbidities	ID	GDD, ID	GDD, ID
Epilepsy diagnosis	Focal epilepsy	LGS	LGS
EEG	Multifocal sharps	GSSW	GSSW, multifocal sharps
ASMs	VPA, LEV, CBZ	VPA, CBD, CLB, LEV	LEV, RUF, LTG, ZNS
Age at primary L-VNS implantation (yrs)	41	20	3
Age at R-VNS implantation (yrs)	45	36	16
Reason for R-VNS	Left extensive scarring	infection	infection
Duration of L-VNS (yrs)	3	3	13
Duration of R-VNS (yrs)	8	6	3.5
Max output current of L-VNS (mA)	1.75	1.75	2.25
Max output current of R-VNS (mA)	1.625	1.375	2.25
Max duty cycle of L-VNS	25%	10%	36%
Max duty cycle of R-VNS	16%	10%	41%
Outcome of L-VNS	IIA	IIA	IIA
Outcome of R-VNS	IA	IIIA	IIIA
Complications from L-VNS	Lead breakage	Lead breakage, infection	Infection
Complications from R-VNS	Transient right neck discomfort	Cyanosis during GTC with a higher setting	None

## Discussion

There are limited studies about the safety and efficacy of R-VNS in human subjects. Cardiac branches from the left vagus nerve modulate the activity of the atrioventricular node, while the branches from the right vagus nerve innervate the sinoatrial node. Stimulation of the sinoatrial nodes is associated with a higher risk of bradycardia, asystole, or other cardiac complications [7]. Therefore, the left vagus nerve is the preferred stimulation site. R-VNS is normally avoided, even though right- and left-sided stimulation demonstrated similar efficacy in suppressing seizures in animal models [12]. In humans, the electrodes in the VNS system are advised to be applied to the vagus truck, which is devoid of any branches [13]. The stimulation of the vagus trunk is generally considered to be safe, with a low rate of adverse cardiac side effects.

There are case reports of nine patients who had R-VNS for the treatment of DRE in the literature [8-10,14,15]. All the reported patients experienced technical difficulties with the left VNS before the R-VNS system was implanted. In a follow-up period ranging from three to 48 months, none displayed severe cardiac complications except one who experienced nocturnal asymptomatic sinus bradycardia [9]. Two pediatric patients had respiratory events with R-VNS [8]. All patients, except two patients whose responses were not documented, demonstrated decreased seizure frequency with R-VNS ranging from 25% to 100%. One patient became seizure-free on R-VNS monotherapy. A different response to seizure control between R-VNS and L-VNS was seen in some patients.

Similar to previous reports, all three patients in our study showed a response to R-VNS therapy. The first patient became seizure-free from her R-VNS, which was not achieved from her L-VNS. The second patient had a weaker response to R-VNS. This may partially relate to his poor tolerance to higher output current, which caused prolonged ictal cyanosis. Seizure-induced cardiovascular and pulmonary dysfunction, together with postictal depression of autonomic respiratory reflexes, likely contribute to ictal cyanosis [16]. R-VNS may worsen this process by triggering bradycardia. His ictal cyanosis returned to baseline after decreasing the output current to 1.375 mA. However, the effect of his VNS on seizure control was also reduced. A previous study showed output current ranging from 1.5 to 1.75 mA is associated with the highest responder rate [17]. Nevertheless, he eventually has good seizure control with a combination of R-VNS and additional epilepsy surgery. The third patient also had a weaker response to R-VNS, even though he was able to tolerate high-intensity settings with R-VNS. We followed these patients for up to eight years and did not appreciate any cardiac or respiratory complications.

It is unclear why some patients had different responses to L- and R-VNS. The exact mechanism of VNS therapy for DRE is also not fully understood. It is proposed that VNS sends electric signals to the nucleus tractus solitarius and other nuclei in the brain stem, which then project diffusely to cortical and subcortical regions including the thalamus, somatosensory cortex, anterior cingulate, and prefrontal cortex [18]. Through these pathways, VNS can modulate neural circuits involved in seizure generation and propagation, leading to the desynchronization of seizure activity [6]. Anatomic and functional variations along these pathways may contribute to the different responses to L-VNS and R-VNS. It is also possible that R-VNS activates different pathways to modulate the epileptogenic network. More research is needed to clarify the underlying etiology.

## Conclusions

Our study demonstrated that R-VNS is a well-tolerated and safe procedure with long-term follow-up. No significant cardiac complications were seen intraoperatively or postoperatively. Side effects can be mitigated by decreasing stimulation intensity. Individual responses vary. The efficacy may be superior or inferior to that of L-VNS. When L-VNS is not technically feasible, R-VNS should be considered with close monitoring for cardiac and respiratory complications. Further clinical trials with large sample sizes are needed to further evaluate the efficacy and safety of R-VNS.
